# Modelling and measurement of laser-generated focused ultrasound: Can interventional transducers achieve therapeutic effects?

**DOI:** 10.1121/10.0004302

**Published:** 2021-04-20

**Authors:** Esra Aytac-Kipergil, Adrien E. Desjardins, Bradley E. Treeby, Sacha Noimark, Ivan P. Parkin, Erwin J. Alles

**Affiliations:** 1 Department of Medical Physics and Biomedical Engineering, University College London, Malet Place Engineering Building, London WC1E 6BT, United Kingdom; 2 Department of Chemistry, Materials Chemistry Research Centre, University College London, 20 Gordon Street, London WC1H 0AJ, United Kingdom

## Abstract

Laser-generated focused ultrasound (LGFU) transducers used for ultrasound therapy commonly have large diameters (6–15 mm), but smaller lateral dimensions (<4 mm) are required for interventional applications. To address the question of whether miniaturized LGFU transducers could generate sufficient pressure at the focus to enable therapeutic effects, a modelling and measurement study is performed. Measurements are carried out for both linear and nonlinear propagation for various illumination schemes and compared with the model. The model comprises several innovations. First, the model allows for radially varying acoustic input distributions on the surface of the LGFU transducer, which arise from the excitation light impinging on the curved transducer surfaces. This realistic representation of the source prevents the overestimation of the achievable pressures (shown here to be as high as 1.8 times). Second, an alternative inverse Gaussian illumination paradigm is proposed to achieve higher pressures; a 35% increase is observed in the measurements. Simulations show that LGFU transducers as small as 3.5 mm could generate sufficient peak negative pressures at the focus to exceed the cavitation threshold in water and blood. Transducers of this scale could be integrated with interventional devices, thereby opening new opportunities for therapeutic applications from inside the body.

## I. INTRODUCTION

Laser-generated focused ultrasound (LGFU) transducers use optically absorbing materials to produce ultrasound via the photoacoustic effect.^1^ LGFU transducers have been used for various therapeutic applications, such as micro-scale fragmentation of solid materials, disruption of cells, drug delivery, and ablation of soft tissue, where the transducers ranged between 6 and 15 mm in diameter (Table I).^1–4^ Such lateral dimensions prohibit employment of LGFU transducers into interventional devices with small channels (<4 mm); therefore, further miniaturization is required. However, the focal gain of a LGFU transducer reduces with decreasing diameter (for a fixed radius of curvature), thereby limiting the maximum achievable pressure. The reduced focal gain can only partially be compensated by increasing the optical pulse energy as this can damage the light-absorbing component. Hence, the question remains, can transducers with dimensions suitable for interventional use generate sufficient pressure at the focus to enable therapeutic effects?

**TABLE I.** Reported characteristics of LGFU transducers used for therapeutic applications. *D*, the diameter of the transducer; *R_c_*, the radius of the curvature; $P_{+}$, peak positive pressure; $P_{-}$, peak negative pressure. The $P_{-}$ values are estimated.

	*D*	*R_c_*	$P_{+}$	$P_{-}$
Reference	(mm)	(mm)	(MPa)	(MPa)
Baac *et al.*,^1^ Baac *et al.*^2^	6	5.5	>50	>25
Di *et al.*^3^	12	12.4	14.5	8
Lee *et al.*^4^	15	9.2	>70	>35

To answer this question, in this paper, we present a simulation model and a measurement study to predict the performance of LGFU transducers for a range of illumination scenarios. We fabricate several transducers^5^ and validate the model against experiments.

Correctly defining acoustic source terms is a crucial part of numerical simulation in ultrasound, particularly in therapeutic ultrasound, to predict potential biological effects in tissue. In previous modelling studies, the pressure field from LGFU transducers was simulated by using a finite element solver^6^ or obtaining approximate analytical solutions for the linear wave equation.^7^ However, these studies have only considered uniform optical fluences across the transducer surface. On the other hand, due to the curved geometry of LGFU transducers, such a distribution is unrealistic and can result in overestimating the generated pressure. The model presented here allows for radially varying fluence distributions and can, thus, accommodate different illumination geometries, including collimated beams and diverging light beams resulting from fibre-optic light delivery, as is routinely used in interventional instruments.

Simulating ultrasound fields generated by LGFU transducers serves several purposes. First, it allows purely numerical investigations into the effects of different LGFU transducer geometries and illumination scenarios. Second, it allows for the accurate extrapolation of acoustic measurements performed at low optical fluence with low-pressure generation to higher pressure levels. This extrapolation enables the use of sensitive calibrated needle hydrophones without risking damage to both the sensor and transducer.^8^ Furthermore, the acquisition of numerous pressure waveforms and characterization of the emitted ultrasound fields over a range of laser power outputs is elaborate experimentally as multiple scans of a hydrophone throughout a three-dimensional (3D) volume are required.^9^ Third, acoustic fields generated by LGFU transducers have, to date, only been measured using water as a medium.^1,10–13^ Through simulations, their performance in other media, such as blood or soft tissue, can be assessed more readily than through *in vitro* and *in vivo* experiments.

This study explores the miniaturization of LGFU transducers to achieve compatibility with minimally invasive medical devices for therapeutic applications. The simulations' results are compared with experiments to test their accuracy, understand the impact of the parameters on the pressure yield, and predict the achievable pressures. The comparisons are made for a range of geometries, illumination scenarios, and both linear and nonlinear regimes. An alternative illumination paradigm is investigated with an aim to increase the efficiency of the transducers.

## II. SIMULATIONS

### A. Simulation model

Acoustic field simulations were performed using version 1.3 of the open-source *k*-Wave matlab toolbox.^14^ The fields were calculated both with and without, including the effects of nonlinear wave propagation. The *k*-Wave toolbox uses a pseudo-spectral time-domain method to solve coupled equations equivalent to a generalised form of the Westervelt equation and can accurately model the nonlinear propagation of transducers with an arbitrary *f*-number ($f_{N}$, $f_{N}=R_{c}/D$).^14,15^ For an accurate representation of the curved source distribution of LGFU transducers on a regular grid, the source geometry was discretised using a discrete band limiting convolution.^16^ The resulting “off-grid” sources avoid staircasing artefacts that occur when source distributions do not coincide with the grid points.

A half-cycle tone burst was used as an excitation signal. Prior to simulations, a measurement of the acoustic field generated by a LGFU transducer under linear conditions in water was performed to derive the source bandwidth with a matched centre frequency to the experiments.

The grid size and number of time steps were varied according to the transducer's dimension, but the Courant–Friedrichs–Lewy number was kept fixed at 0.3 for all simulations.^17^ A perfectly matched layer was imposed on a 20 grid-point-thick layer at each of the computational domain edges. The grid spacing was determined using 2 points-per-wavelength (the Nyquist limit) at the maximum supported frequency set to 50 MHz for linear propagation and 75 MHz for nonlinear wave propagation to support higher harmonics (This corresponds to approximately ten harmonics for a centre frequency of 7 MHz and five harmonics at the highest centre frequency of 13.4 MHz). The speed of sound, $c_{0}$, and the density of the medium, $\rho_{0}$, were set to those of water as 1480 m/s and 1000 kg/m^3^, respectively.^18^ The attenuation coefficient of water was modelled by a power law of the form $\alpha_{0}f^{b}$ where $\alpha_{0}=0.00217\;\;\text{dB}\;\;{\text{cm}}^{-1}\;\;{\text{MHz}}^{-b}$ and *b* = 2.^19^ The nonlinearity parameter for water (B/A=5) was added to the medium properties when nonlinear propagation was considered.^20,21^ The simulated time-varying pressures were recorded throughout the simulation grid.

A two-dimensional (2D) axisymmetric model was used to decrease the computational complexity of the simulations. A half arc source was defined within an axisymmetric coordinate system to represent the transducer's concave geometry in two dimensions.^15^ The integration points were located at the support of the true source and spread equally over the arc. The number of integration points was calculated by upsampling the equivalent number of grid points over the arc length by four. For each point given in the arc, a band limited interpolant was computed, corresponding to a point source at that location. The point sources were then summed to provide the source mask. The magnitudes of the individual interpolants were scaled according to the radially varying fluence distributions, which will be explained in Sec. II B.

### B. Modelling fluence distribution on the surface of a LGFU transducer

The typical assumption of uniform fluence distribution on the transducer surface is very challenging to obtain due to the geometrical differences between the light beam and transducer surface. For this reason, the fluence distribution was allowed to vary radially to take these differences and the fluence nonuniformities of the light into account. To include common strategies to illuminate LGFU transducers, we considered two ways that the excitation light can reach the transducer surface: (a) collimated beam delivery through free-space optics or via a fibre collimator and (b) diverging light delivery via an optical fibre. The first illumination strategy (“collimated”) assumes that the incident beam is to be collimated with a top-hat beam profile, resulting in a radially varying fluence across the transducer surface. The second illumination strategy (“diverging”) models the light delivery through an optical fibre and assumes the light is delivered from a point and propagates in a conical fashion. For both cases, the laser beam profile was assumed to be top-hat; thus, the fluence of the laser beam was considered radially invariant.

**FIG. 1.**
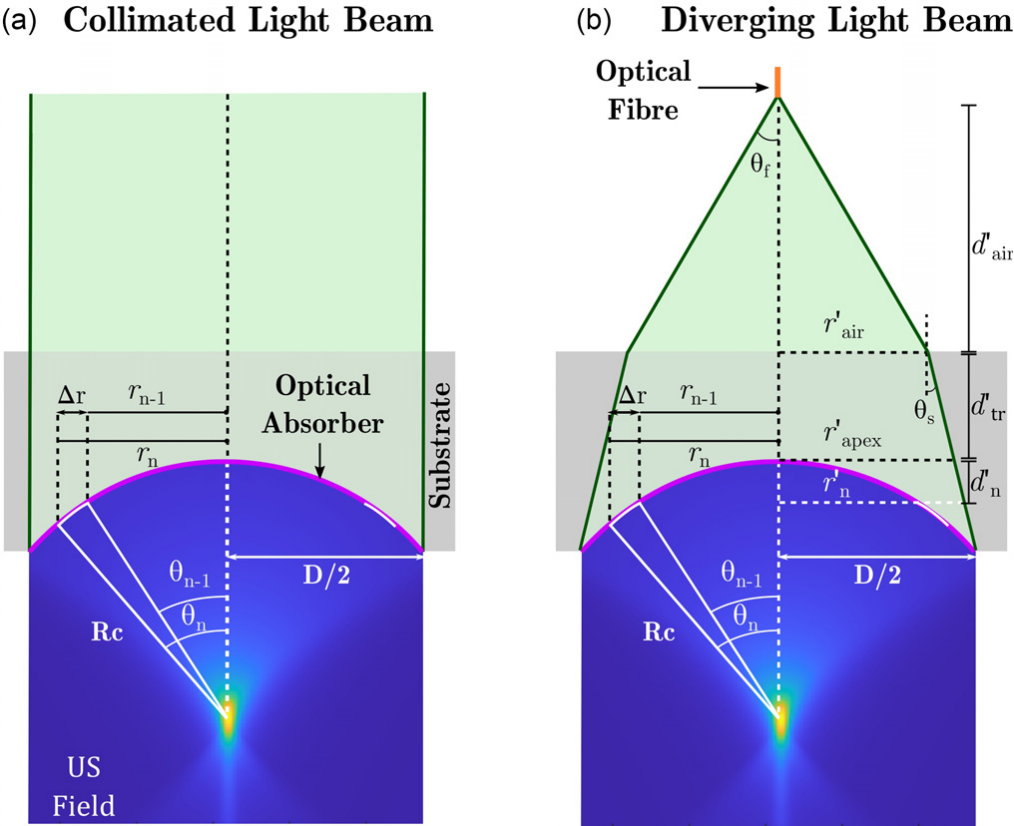
(Color online) Schematics of the fluence distribution model on the surface of a transducer for two cases. (a) Collimated light from a free-space beam or a collimator and (b) diverging light from an optical fibre are shown. *R_c_*, the radius of curvature; *D*, the diameter of the transducer; US, ultrasound; $\theta_{n}$, polar angle corresponding to the *n*th ring; $\theta_{a}$, divergence angle.

Figure 1 illustrates how the fluence at the transducer surface was modelled. For the collimated light delivery case [Fig. 1(a)], the light beam was divided into a set of equidistantly spaced concentric rings with an outer radii of *r_n_* with respect to the optical axis and a width of Δr. The spherical surface of the transducer was divided into the annular projections of these rings. The resulting fluence was weighted according to the ratio between the areas of the *n*th ring of the laser beam and corresponding *n*th annulus along the transducer surface.

The area of the *n*th ring of the laser beam was calculated by subtracting the area of the (*n* – 1)th disk from the area of the *n*th. The areas of the annuli on the transducer surface were obtained by evaluating the surface integral along the spherical transducer surface between the polar angles of $\theta_{n-1}$ and $\theta_{n}$.^22^ Therefore, the fluence weighting function for the *n*th annulus, $W_{\text{coll},n}$, was expressed as

$$W_{\text{coll},n}=\frac{\left[{\left(n\Delta r\right)}^{2}-{\left((n-1)\Delta r\right)}^{2}\right]}{-2R_{c}^{2}[\text{cos}(\theta_{n})-\text{cos}(\theta_{n-1})]},$$ (1)

where *R_c_* is the radius of the curvature of the transducer. The angles were attained from the azimuthal projection of the radii of the rings $r_{n-1}$ and *r_n_* as $\theta_{n-1}=\text{arcsin}(((n-1)\Delta r)/R_{c})$ and $\theta_{n}=\text{arcsin}((n\Delta r)/R_{c})$, respectively.

In the diverging illumination case, the beam radius increases over the propagation distance from the first to the last annulus [Fig. 1(b)]. The expansion of the beam toward the edges causes a reduction in the fluence with depth. The resulting fluence decrease weighting function for the *n*th annulus, $W_{\text{div},n}$, was calculated as the ratio of the area of the beam at the apex of the transducer surface comprising an optically absorbing layer to the area of the beam at the location of the *n*th annulus,

$$W_{\text{div},n}=\frac{{\left({r^{\prime }}_{\text{apex}}\right)}^{2}}{{\left({r^{\prime }}_{n}\right)}^{2}}.$$ (2)

For the collimated case, $W_{\text{div},n}$ is equal to one for all *r_n_*.

The radius of the illumination beam at the level at the apex of the spherical surface, ${r^{\prime }}_{\text{apex}}$, was determined by the optical fibre used to deliver the laser output (FG200LCC, 0.22 NA, 200 *μ*m core diameter, Thorlabs, Bergkirchen, Germany). Likewise, the divergence angle, $\theta_{a}$, was obtained from the numerical aperture of the fibre in air. Upon propagation across the air–substrate interface [Fig. 1(b)], the excitation light refracts due to a mismatch in the refractive indices and travels with the angle of incidence of $\theta_{s}$ within the substrate, which was determined by Snell's law. It was assumed that the beam diverges exactly up to the transducer's outermost edge, i.e., the aperture of the transducer was not overfilled. Thus, ${r^{\prime }}_{\text{apex}}$ was calculated with the following equation:

$${r^{\prime }}_{\text{apex}}=\frac{D}{2}-\left(R_{c}-\sqrt{R_{c}^{2}-{\left(\frac{D}{2}\right)}^{2}}\right)\text{tan}(\theta_{s}).$$ (3)

The radius of the beam at the *n*th intersection plane with the transducer surface, ${r^{\prime }}_{n}$, was calculated as

$${r^{\prime }}_{n}={r^{\prime }}_{\text{apex}}+\left(R_{c}-\sqrt{R_{c}^{2}-{\left(n\Delta r\right)}^{2}}\right)\text{tan}(\theta_{s}).$$ (4)

The distance between the fibre tip and transducer, ${d^{\prime }}_{\text{air}}$, required to achieve edge-to-edge illumination was obtained from

$${d^{\prime }}_{\text{air}}=\frac{{r^{\prime }}_{\text{air}}}{\;\text{tan}(\theta_{a})},$$ (5)

where ${r^{\prime }}_{\text{air}}$ is the radius of the beam at the air–substrate interface and was attained using the formula

$${r^{\prime }}_{\text{air}}={r^{\prime }}_{\text{apex}}+{d^{\prime }}_{\text{tr}}\;\text{tan}(\theta_{s}).$$ (6)

The minimum thickness of the transducer, ${d^{\prime }}_{\text{tr}}$, was kept fixed for all diameter values for a given radius of curvature.

The total fluence weighting function for the *n*th annulus, $W_{\text{tot},n}$, was calculated based on the combined effect of (i) the geometrical differences between the laser light and transducer surface and (ii) the optical fluence decrease caused by the beam divergence over the propagation distance along the transducer surface as

$$W_{\text{tot},n}=W_{\text{coll},n}\times W_{\text{div},n}.$$ (7)

**FIG. 2.**
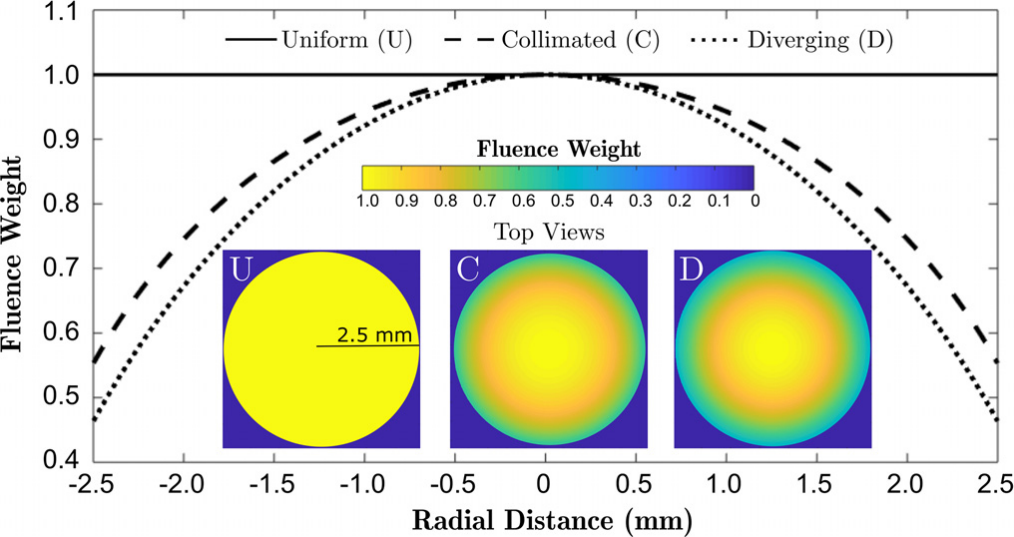
(Color online) Normalised fluence weights versus the radial distance from the centre of a transducer for the uniform case (*U*) and the nonuniform cases resulting from collimated (*C*) and diverging (*D*) light delivery (*R_c_* = 3, *D* = 5 mm). The insets show the top-down projections of the spatially varying fluence distributions across the surface of the LGFU transducer.

The weighting coefficients were normalised to the unit value at the apex of the spherical section for both of the light delivery cases. As an illustration, Fig. 2 shows the surface fluence distributions for a transducer with a radius of curvature of 3 mm and diameter of 5 mm.

Radially varying fluence distributions [Eq. (7)] and the corresponding initial acoustic source distributions were implemented into the simulation model explained in Sec. II A to account for these two realistic light delivery scenarios.

## III. EXPERIMENTAL SETUPS

Experimental setups were constructed for three cases: collimated light delivery from a free-space beam, diverging light delivery from an optical fibre, and inverse Gaussian beam delivery. As uniform illumination across the curved transducer surface is not easily realised in practice, it was not considered.

### A. Transducer fabrication

LGFU transducers with two different geometries, (I) *R_c_* = 3, *D* = 5 mm and (II) *R_c_* = 5, *D* = 9 mm, were fabricated incorporating gold nanoparticles (AuNPs)-polydimethylsiloxane (PDMS) and multiwalled carbon nanotubes (MWCNTs)-PDMS composites, respectively.^5^

### B. Light delivery

#### 1. Collimated case

**FIG. 3.**
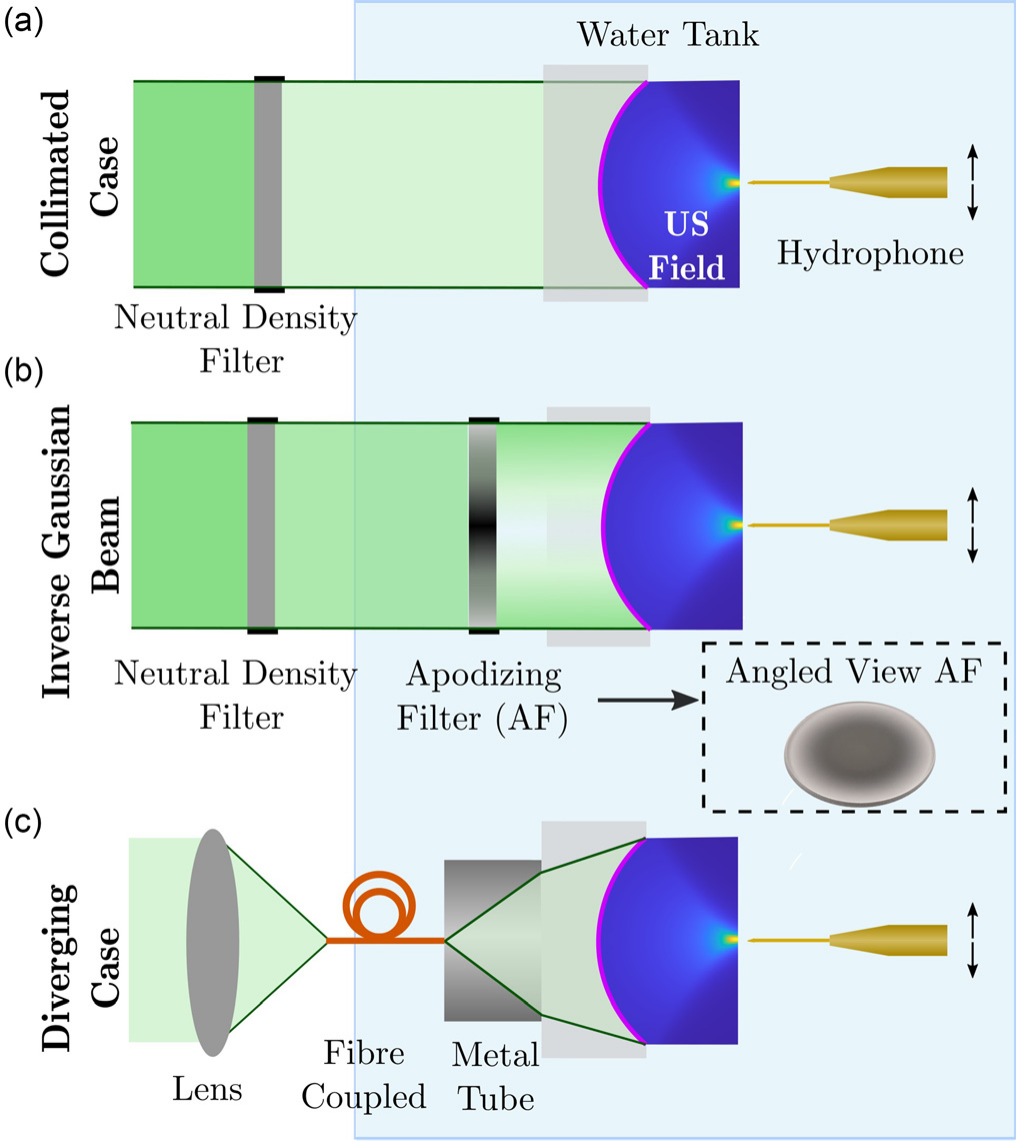
(Color online) Schematics of the experimental setups with different excitation schemes. (a) The collimated case, (b) inverse Gaussian beam, and (c) diverging case are shown. At high fluence levels (>4 mJ/cm^2^), a custom fibre-optic hydrophone (FOH) and at low fluence levels (<4 mJ/cm^2^), a commercial needle hydrophone is used as a sensor. The hydrophone was translated across a 2D plane.

As an excitation source, a pulsed *Q*-switched Nd:YAG laser (Quanta-Ray, INDI-40–10, Spectra-Physics, Santa Clara, CA; wavelength, 532 nm; pulse duration, 6 ns; pulse repetition frequency, 10 Hz) was used. The LGFU transducer was mounted in a glass tank filled with degassed, de-ionized water, and the collimated output of the laser was coupled to it in free-space [Fig. 3(a)]. The pulse energy was adjusted by neutral density filters (Comar, Surrey, UK) placed between the laser and the transducer.

#### 2. Inverse Gaussian beam

The excitation light source generates an approximately near top-hat beam, which results in a radial drop-off of the fluence distribution toward the edges of the transducer caused by the geometrical differences between the laser light and its surface area [Eq. (7)]. To partially compensate for this, a second illumination strategy was considered in which an apodising neutral density filter (NDY20A, Bergkirchen, Germany) was placed in the light path [Fig. 3(b)].

#### 3. Diverging case

In clinical practice, fibre-coupled lasers are envisioned due to their greater practicality. To study this scenario, as an excitation source, a pulsed laser (FQ-200–20-V-532, Elforlight, Northants, UK; wavelength: 532 nm, pulse duration: 10 ns, pulse repetition frequency: 100 Hz) with a maximum pulse energy of 40 *μ*J was used. The fibre-coupled output of the laser was paired with the transducer mounted within a section of metal tubing. A fibre chuck integrated with the tubing was used to coaxially deliver the light to the transducer [Fig. 3(c)].

### C. Acoustic measurements

At low fluence values (<4 mJ/cm^2^), the acoustic pressure field was measured with a needle hydrophone (75 *μ*m; Precision Acoustics, Dorchester, UK; calibrated within the range from 1 to 30 MHz) positioned with a three-axis computer-controlled motorized translation stage (MTS50/M-Z8, Thorlabs, Bergkirchen, Germany). This hydrophone was positioned at the geometrical focus of a LGFU transducer; its signal was pre-amplified by 20 dB (DHPVA-200, Femto, Germany), digitized (14 bits, 125 MS/s, M4i.4420-x8, Spectrum, Munich, Germany), and stored. At high fluence levels (>4 mJ/cm^2^), a custom fibre-optic hydrophone (FOH) comprising a bare, flat-cleaved single-mode optical fibre (core diameter 9 *μ*m) was used due to its higher resilience against high ultrasound pressures.^1^ The FOH was probed at 1530 nm using a continuous wave laser (1500–1600 nm; TUNICS T100S-HP/CL, Yenista Optics, Lannion, France), operating at 24 mW. An optical circulator was used to deliver the light to the fibre's tip and return the reflected light to a photodiode (DET01CFC, Thorlabs, Bergkirchen, Germany). The photodiode output was pre-amplified by 60 dB, digitized, and used to record the reflected optical power modulation resulting from refractive index changes of the surrounding water, generated by the incident ultrasound wave.

For the collimated light delivery case, either the needle hydrophone or FOH were used to measure the generated ultrasound pressure amplitudes and bandwidths, depending on the fluence. In the cases of an inverse Gaussian beam profile and diverging light delivery, a low fluence was used due to the low damage threshold of the apodising filter (25 mJ/cm^2^) and light source's limited capacity, respectively. For this reason, in these scenarios, the needle hydrophone was used as a sensor.

## IV. LINEAR ACOUSTIC FIELDS—MODEL SIMULATIONS AND VALIDATION MEASUREMENTS

Several experiments were conducted in a range of illumination scenarios to validate the numerical model with radially-varying acoustic input distributions.

### A. Model versus experiment—Collimated and diverging cases

For the collimated light delivery case, the acoustic field generated by a LGFU transducer with an outer diameter of 5 mm and radius of curvature of 3 mm was propagated in water. Simulations were run on a computational grid comprising 216 × 324 elements. The temporal step was 3 ns, and the simulations were performed for 1202 time steps. Similarly, for the diverging light delivery case, the acoustic field generated by a LGFU transducer with a diameter of 9 mm and radius of curvature of 5 mm was simulated in water. All of the simulation parameters were the same for these transducers (Sec. II A), except the grid size and time step numbers, which in the latter case were 360 × 576 and 2003, respectively.

**FIG. 4.**
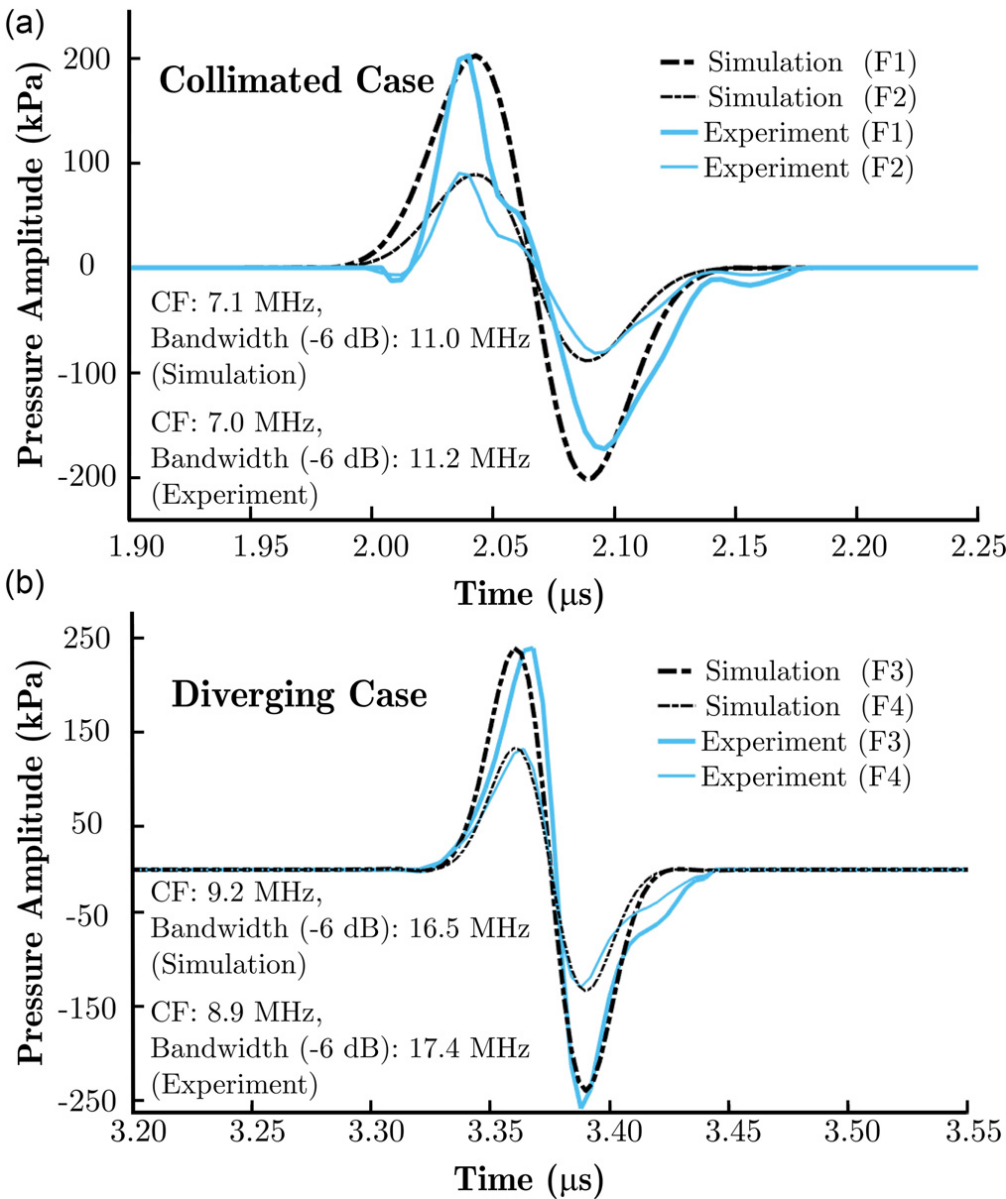
(Color online) (a) Simulated and measured ultrasound pressure waveforms in the collimated light delivery case for two different fluence values, F1=0.34 mJ/cm^2^ and F2=0.16 mJ/cm^2^ (*R_c_* = 3, *D* = 5 mm; AuNPs-PDMS), (b) Simulated and measured ultrasound pressure waveforms in the diverging light delivery case for two different fluence values, F3=0.17 mJ/cm^2^ and F4=0.09 mJ/cm^2^ (*R_c_* = 5, *D* = 9 mm; MWCNTs-PDMS). CF, centre frequency.

The measurements were performed in water with the setups explained in Sec. III for two different fluence values. The waveforms in Fig. 4 result from averaging ten signal traces. Peak positive and peak negative pressures and the shapes of the ultrasound waves observed at the position of maximum positive pressure were used as metrics for comparison between the simulations and experiments. A measurement of the acoustic field generated by a LGFU transducer at the lower fluence, which satisfies linear conditions, was used to (i) determine the bandwidth and centre frequency of the excitation signal in the simulations, and (ii) match the peak positive pressures of the simulated and measured acoustic waves. In the simulations, according to the fluence distribution model, a radially varying surface pressure distribution was initially assigned with a maximum amplitude of 1 MPa, and the acoustic field was propagated. The maximum surface pressure amplitude (not explicitly known) was then adjusted to match the peak positive pressure of the simulated wave with the measured wave at the focus at the lower fluence. At the higher fluence, the source distribution was scaled according to the fluence ratio between the measurements. The simulated acoustic wave was compared with the corresponding experiment. The centre frequencies of the transducers used in the collimated and diverging light delivery cases were measured to be approximately 7.1 and 9.1 MHz, respectively.

For the collimated case [Fig. 4(a)], the measured peak negative pressure differs from the simulated value by 8% and 14% for fluences of 0.16 and 0.34 mJ/cm^2^, respectively. The optical energies were measured at the end of the fibre output of the laser with a thermopile head (818 P-001–12, MKS/Newport Corporation, Irvine, CA) connected to an optical power meter (1918-R, MKS/Newport Corporation, Irvine, CA). Neutral density filters were used to achieve these fluences. For the diverging case [Fig. 4(b)], the measured peak negative pressure differs from the simulated value by 5% and 7% for fluences of 0.09 and 0.17 mJ/cm^2^, respectively. These differences are close to the previously reported values for the expected uncertainty of the ultrasound pressure measurement;^23^ thus, there is good agreement between the simulated and measured pressure waves. Comparing Figs. 4(a) and 4(b), the collimated case shows a greater discrepancy between the simulation and experiment. This deviation could possibly arise due to microstructural differences in the material composites, the inherent variability of manual fabrication steps, or a variation in the beam profiles of the two excitation sources.

### B. Model versus experiment—Inverse Gaussian beam

The apodising filter was used in the experimental setup to achieve an approximately inverse Gaussian beam. In the model, the apodising filter was incorporated by an additional term provided by the manufacturer in Eq. (7).

The acoustic field generated by a LGFU transducer with an outer diameter of 9 mm and radius of curvature of 5 mm was simulated in water. The same grid parameters that were described for the diverging case were used in the simulations. The initial source distribution was adjusted for the simulations such that the total energy of the laser light was equal for the apodised and non-apodised cases.

As an ultrasound generator, a different transducer with an aperture diameter of 9 mm and radius of curvature of 5 mm was used. The centre frequency of the transducer was measured to be approximately 6.7 MHz. For the first set of experiments, a stack of neutral density filters with a combined optical density value of 2.5 was used. A custom holder was used to coaxially align the filters with the transducer. For the second set of experiments, the optical density value was decreased to 1.5 to achieve a similar signal-to-noise ratio (SNR) to the initial measurements with a radially varying optical density from 0.04 to 2 (edge-to-centre). In the first set of experiments, the numerically integrated total energy of the light beam was 2.5 times higher than it was in the second set. This factor was corrected numerically in post-processing. The excitation light fluence was kept below the damage threshold of the apodising filter, which was listed as 25 mJ/cm^2^ (at 532 nm, 10 ns, 10 Hz). In principle, this limitation could be overcome by developing a custom apodising hard dielectric coated filter with a much higher damage threshold (e.g., 2 J/cm^2^ versus 25 mJ/cm^2^ at 532 nm, 10 ns, 10 Hz).

**FIG. 5.**
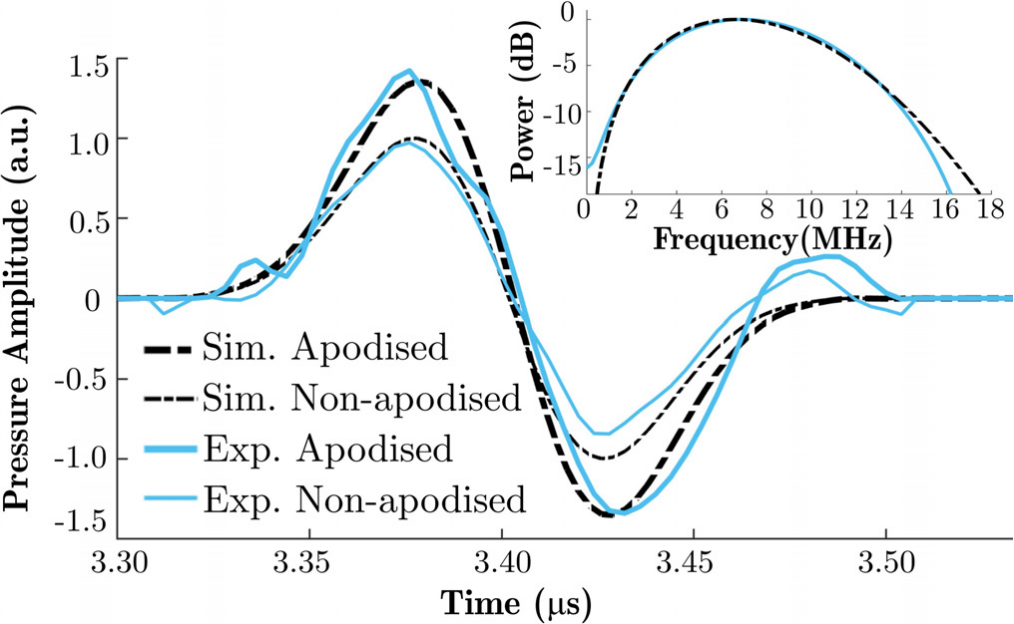
(Color online) Pressure waveform comparison between the experiments and simulations for apodised and non-apodised cases. (Inset) Ultrasound power spectra comparison between the experiments and simulations (Transducer, *R_c_* = 5, *D* = 9 mm; MWCNTs-PDMS). CF, centre frequency; CF = 6.6 MHz, bandwidth (–6 dB), 10.3 MHz (simulation); CF = 6.8 MHz, bandwidth (–6 dB), 10.5 MHz (experiment).

For the same total light energy, the pressure amplitude at the focus was observed to be 35% higher when the apodising filter was present (Fig. 5). These observations agree very well with the simulations. A higher pressure was measured for the inverse Gaussian beam profile as, in this case, a higher fluence was delivered to the outer edge of the transducer, which contributed to a higher gain factor.

### C. The effect of nonuniform fluence distributions

**FIG. 6.**
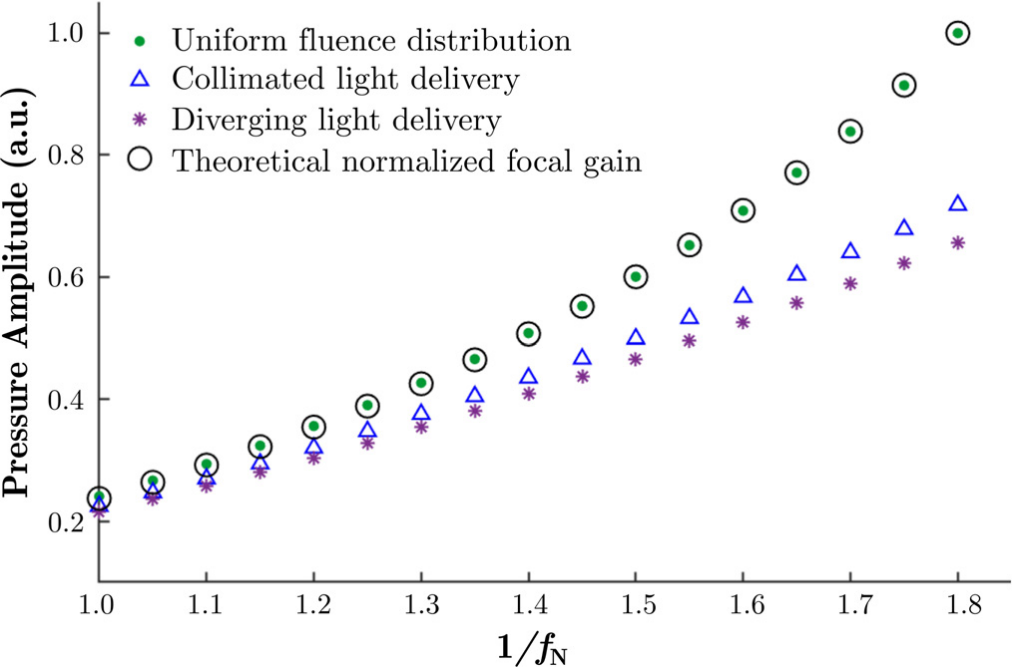
(Color online) Theoretical focal gain and pressure amplitudes resulting from hypothetical uniform and nonuniform fluence distributions normalised to the maximum gain at $1/f_{N}=1.8$.

Linear simulations in absorbing water were performed to propagate ultrasound fields generated by LGFU transducers with various geometries. Pressure fields were acquired for transducers with diameters ranging from 1.0 to 1.8 times their radius of curvatures. The theoretical estimations of the focal gains of the LGFU transducers were normalised to the maximum gain at a $1/f_{N}$ ratio of 1.8. Numerically obtained focal pressures resulting from hypothetical uniform and nonuniform fluence distributions for various $1/f_{N}$ ratios were normalised and compared to the maximum gain (Fig. 6). For the case of the uniform fluence distribution, the pressure in the focal region agrees very well with that predicted by the theoretical focal gain. However, for the more realistic cases of collimated or diverging illumination, the pressure amplitude at the geometrical focus reduced by up to 35% and 45%, respectively. In the linear regime, these results were observed to be independent of the radius of curvature provided the same $f_{N}$ (data are not shown). When the fluence distribution is uniform, the pressure amplitude increases in a quadratic form with $1/f_{N}$. However, for the diverging case, a nearly linear relationship between focal pressure and $1/f_{N}$ was observed.

## V. NONLINEAR ACOUSTIC FIELDS—MODEL SIMULATIONS AND VALIDATION MEASUREMENTS

The second set of nonlinear simulations, including attenuation, was performed to model ultrasound fields from the LGFU transducers with various geometries using homogeneous water as a medium.

To compare the model with experiments in a nonlinear medium, a LGFU transducer with an outer diameter of 5 mm and radius of curvature of 3 mm was used as an acoustic field generator. The maximum frequency was set to 75 MHz to support higher-order harmonics. As a result, the spatial step size was taken as 10 *μ*m (2 points-per-wavelength in water) and, consequently, the temporal step was taken as 2 ns. Simulations were run on a 324 × 512 computational grid with 1803 time steps.

**FIG. 7.**
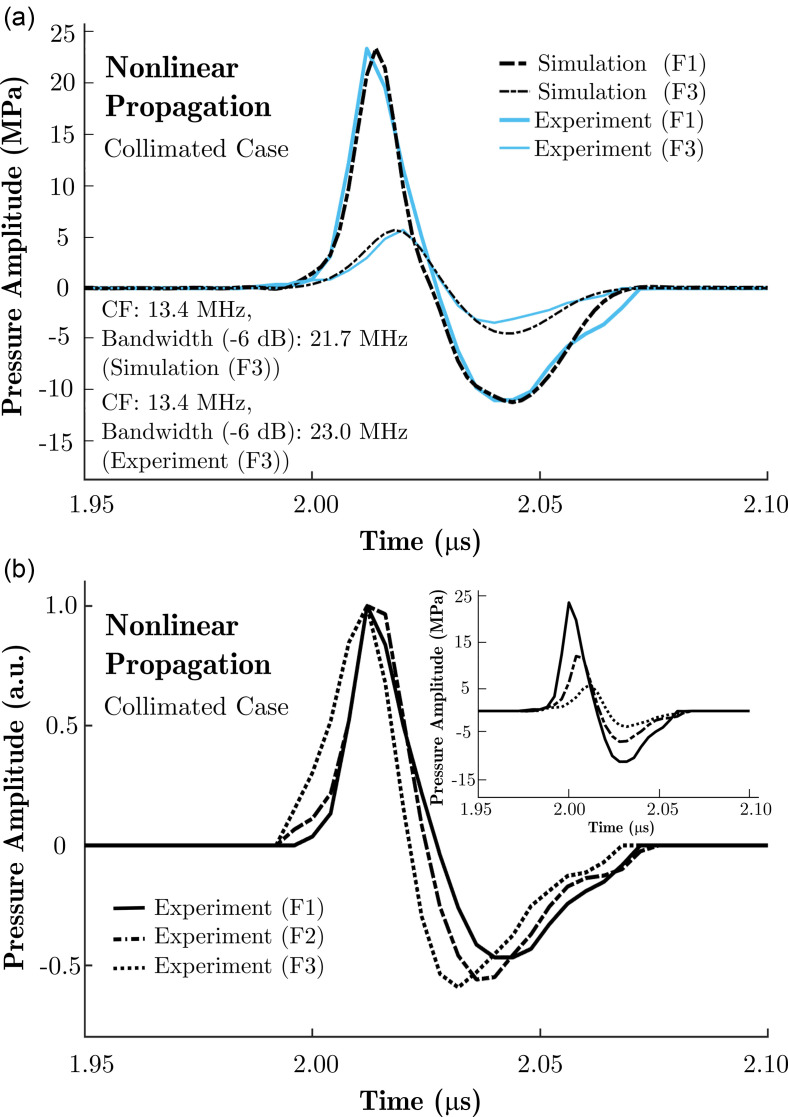
(Color online) (a) Simulated and measured ultrasound pressure waveforms for nonlinear propagation in the collimated light delivery case for two different fluence values. (b) Pressure traces were normalised and delayed to match in arrival time to highlight the wavefront steepening. (Inset) Original pressure traces. F1=38 mJ/cm^2^, F2=24 mJ/cm^2^, and F3=12 mJ/cm^2^ (*R_c_* = 3, *D* = 5 mm; AuNPs-PDMS).

The experimental setup explained in Sec. III B was used with laser fluences >4 mJ/cm^2^. The custom FOH was used as a sensor for the acoustic field measurements. The sensitivity of the FOH was determined to be 6 mV/MPa at low ultrasound pressures by comparing the signal amplitudes with those obtained using the calibrated needle hydrophone and was similar to the sensitivity reported in the study by Baac *et al.*^1^ The measurements were taken along the optical axis at the axial distance, coinciding with the maximum pressure, and at various fluence levels. A series of 1500 pressure waveforms were recorded for each fluence. Pressure waveforms were high-pass filtered using an infinite impulse response Butterworth filter design with a frequency cutoff of 2 MHz (the preamplifier also applied a low-pass filter at 200 MHz). In an acoustic cavitation event, the interrogation light within the FOH reflected from a glass–gas interface rather than a glass–liquid interface, which resulted in a larger refractive index mismatch and, consequently, a larger positive signal that saturated the photodiode. These saturated signals were excluded, and the remaining signals were used for signal averaging. A rectangular temporal window around the ultrasound response was applied to exclude noise. The waveforms acquired at two different fluence levels were plotted for comparison with simulations in Fig. 7(a). The measurement taken at the lowest fluence was used to match the simulations to experiments as explained in Sec. IV A. The centre frequency of the transducer was measured to be approximately 13.4 MHz.

A good agreement was observed between the measured and simulated data. The measured peak negative pressure differs from the simulated value by <1% and 20% for the laser fluence values of 38 mJ/cm^2^ and 12 mJ/cm^2^, respectively. The greater difference observed for the lower fluence case can be attributed to a lower SNR. The optical energies were measured after the neutral density filters with an absorbing calorimeter (AC2501, Scientech, Boulder, CO) connected to an optical power meter (Vector H410, Scientech, Boulder, CO).

The nonlinear propagation was further exemplified in Fig. 7(b) where experimentally obtained pressure traces were shown for three fluence levels (F1=38 mJ/cm^2^, F2=24 mJ/cm^2^, and F3=12 mJ/cm^2^). The measured acoustic waves were normalised to their positive peaks and shifted in time such that their maxima coincide. Wavefront steepening and a decrease in the arrival time can be observed with increasing fluence, which are signature effects of nonlinear propagation.

## VI. PREDICTING THE PERFORMANCE OF INTERVENTIONAL LGFU TRANSDUCERS

In Secs. II–V, a model for LGFU transducers was developed and validated using experimental measurements. In this section, the developed model was used to explore the question: can transducers with dimensions suitable for interventional use generate sufficient pressure at the focus to enable therapeutic effects?

To predict the achievable pressures from LGFU transducers, the maximum surface pressure was considered as 4 MPa. This boundary condition was chosen to match previously reported values without any observed damage to the coatings.^24^ However, when using the *k*-Wave toolbox in transient (non-pulsed mode), the input is injected as an additive mass source, and a pressure boundary condition is not directly defined. For this reason, to calibrate the source strength at the transducer surface, the following numerical experiment was conducted. 3D simulations were performed in the linear regime with a bowl surface defined as the source geometry ($R_{c}=3$, *D* = 5 mm). The centre frequency of the transient acoustic input was assigned as 10 MHz, which represents the average value of the experimental measurements, and its amplitude was defined as 1 MPa. The pressure field was recorded at a nearby plane from the distal end of the transducer surface to stay away from focus and, hence, stay in the linear regime. The field was numerically backpropagated to different parallel axial planes that were 1 *μ*m apart from each other using the angular spectrum approach.^25^ From the propagated planes, the surface pressure at the apex of the transducer was found to be 1.6 MPa. A scaling factor was added to compensate for the incomplete acoustic capture of the pressure field.^26^ The strength of the tone burst excitation was injected as 2 MPa based on the above numerical experiment and calculations to assign the boundary surface pressure as 4 MPa.

**FIG. 8.**
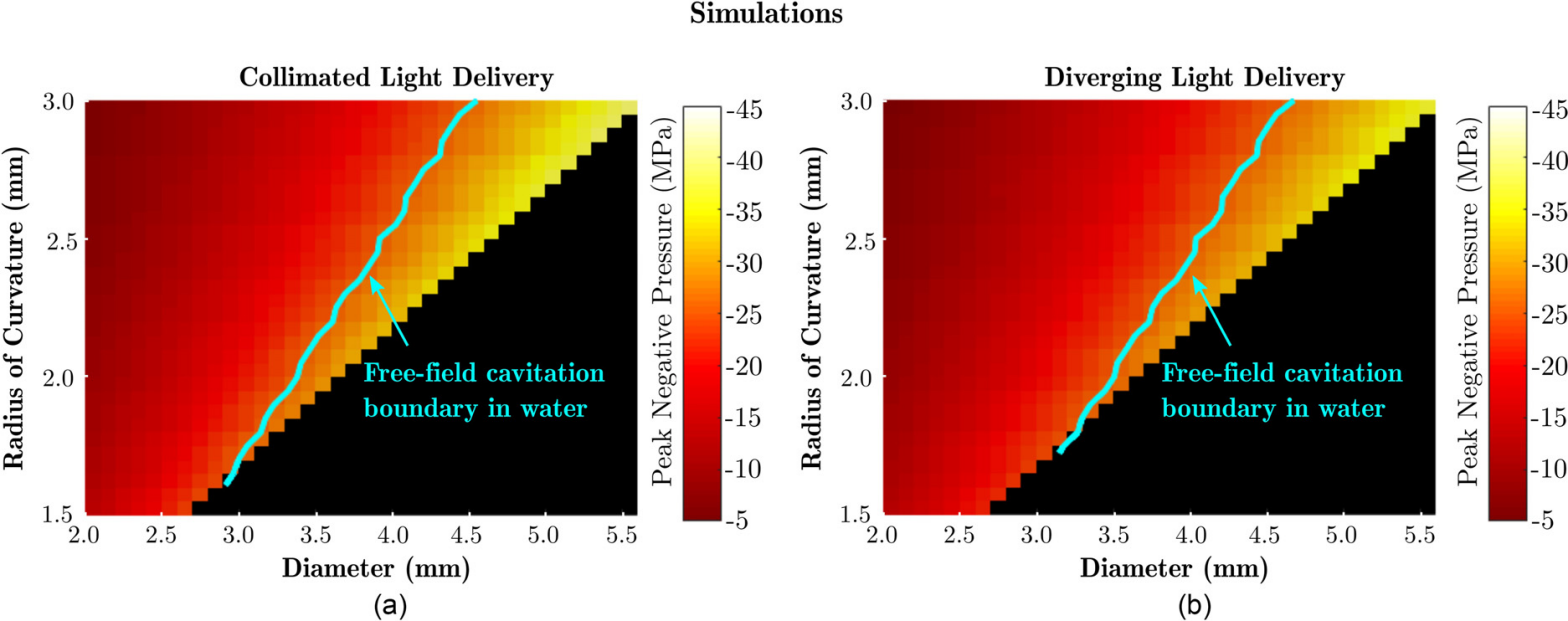
(Color online) Surface plots of peak negative pressures on the geometric focus of transducers for two cases. (a) Collimated light from a free-space beam or a collimator and (b) diverging light from an optical fibre are shown. A contour plot at a negative pressure level required for free-field cavitation in water is superimposed on the surface plots. It should be noted that the peak negative pressure is, in practice, limited by the cavitation threshold of the medium.

Various geometries of the LGFU transducers were explored to determine their capabilities in the achievable pressures at their foci. The diameters and radius of curvatures ranged from 2.0 to 5.6 mm and 1.5 to 3 mm, respectively. Simulations were run in homogeneous water, including nonlinearity and attenuation (Sec. V). The central acoustic frequency was taken as 10 MHz. Figure 8 shows the surface plots of peak negative pressures at the geometric focus of transducers for two cases: (a) collimated light from a free-space beam or a collimator and (b) diverging light from an optical fibre. A contour plot at a negative pressure level required for free-field cavitation in water ($P_{-}$ threshold = –26.2 MPa) is superimposed on the surface plot.^27,28^ As it can be inferred from Fig. 8, cavitation can be generated in free-space in water with a transducer diameter as small as 3.5 mm. This result is consistent with a preliminary study's experiments in which cavitation was achieved on a rigid surface with a LGFU transducer with a 3 mm diameter.^5^ As the highest pressure is achieved near the geometrical focus of the LGFU transducer, the therapeutic working distance is approximately equal to the radius of curvature. As a result, a trade-off between the lateral device dimension and working distance needs to be made for a fixed pressure value suitable for therapy.

## VII. DISCUSSIONS

In this work, we present an efficient numerical model of the LGFU transducers that allows for the modelling of realistic, nonuniform light distributions across the transducer surface. This model is based on an axisymmetric version of *k*-Wave^15^ to reduce the computational complexity, and uses off-grid sources^16^ to avoid staircasing errors. The framework includes linear and nonlinear propagation as well as acoustic attenuation. The model was validated against a series of experimental results and finally used to confirm that the LGFU transducers suitable for interventional use (with diameter<4 mm) are capable of generating sufficient pressure levels to achieve therapeutic effects.

Although high peak pressures of 24.5 MPa/–11.5 MPa (for peak positive and peak negative) were shown (Fig. 7) and cavitation on the tip of the FOH was observed in the experiments for higher fluence values (data not shown), the focal gain of the fabricated LGFU transducers was estimated to be 1.5 times smaller than the theoretical expectations. This is likely due to imperfections in the transducer shape introduced during fabrication. To date, LGFU transducers have been fabricated using two main methods: (i) an optical absorber is deposited onto a concave substrate and subsequently overcoated with PDMS^1–4,10,12^ or (ii) an elastomeric moulding process is used.^5,13^ Both methods allow for variability and irregularities in the transducer geometry. To confirm the negative impact of such irregularities on the focal gain, simulations were performed for LGFU transducers of which the geometry was perturbed by applying randomised height offsets across the transducer surface (assuming radial symmetry; data not shown). When these perturbations were randomly sampled from narrow distributions spanning ±λ/2 (±50 *μ*m) or ±λ (±100 *μ*m), the focal gain was found to decrease by 10% and 25%, respectively. Thus, care is required to ensure the fabricated LGFU transducer surfaces are as close to spherical as possible, ideally within ±λ/2 or better. The observed discrepancy between the theoretical and experimental focal gain complicates the simulation predictions of absolute pressure values and introduces some uncertainty in the pressures predicted in Fig. 8. However, the comparative results presented in the remainder of this work remain valid as the focal gain's influence is removed through experimental calibration.

A decreased performance in the focal gain and corresponding generated focal pressure can be offset in several ways. First, in the presented simulations, the maximum pressure amplitude at the transducer surface was limited to 4 MPa to match previously reported values^24^ and provide a reasonable safety margin. However, studies have reported surface pressures as high as 13.8 MPa without damaging the transducer using similar coating materials.^29^ Significantly higher pressures than those reported here can, therefore, likely be generated by increasing the optical fluence. Nevertheless, in practice, the maximum peak negative pressure is limited by the cavitation threshold of the medium. Second, the pressure generated by a LGFU transducer can be increased through optimisation of the optical fluence distribution. In this work, we showed how a diverging light beam, as delivered by a bare optical fibre, resulted in up to a 45% reduction in the focal pressure compared to uniform illumination. In contrast, light beams of an approximately inverse Gaussian profile generated significantly higher pressures at the acoustic focus (an increase of 35% for the case considered here). In the future, it would be of interest to derive closed-form expressions for the focal gain for a range of different apodisation scenarios. Whereas at present, a low damage threshold of the apodising filter limits the achievable focal pressure, more resilient filters or telescopic setups could be developed to generate therapeutically relevant pressures. Moreover, with an inverse Gaussian illumination pattern, the outer parts of the transducer contribute most to the focal pressure. The centre area could, thus, be sacrificed to integrate an ultrasound detector or deliver an instrument, for instance.^30^

Third, the cavitation threshold of a medium is affected by its purity. In the simulations and experiments in this work, purified and degassed water were considered, and a lack of cavitation nuclei results in a high free-field cavitation threshold. Achieving free-field cavitation in circulating blood was reported to be challenging.^31,32^ However, the cavitation threshold of a medium can be significantly reduced by deliberately introducing impurities in the form of ultrasound contrast agents, hence, providing cavitation nuclei. For instance, introducing phase-shift droplets in tissue-mimicking phantoms reduced the cavitation threshold (at peak negative pressure) from −26.8 ± 0.5 MPa to −14.9 ± 0.4 MPa at a sonication frequency of 3 MHz.^28^ In addition, the numerical studies show that the reduction in the cavitation threshold with the addition of a contrast agent can be further enhanced when the agent's diameter is matched with the centre frequency of the pressure wave.^33^ As LGFU transducers typically operate above 3 MHz, a higher cavitation threshold is foreseen with the increased frequency;^1^ for example, extrapolating the work by Vlaisavljevich *et al.*^28^ to a frequency of 10 MHz, a contrast agent-mediated cavitation threshold of −18.6 MPa could be expected.

In this study, the propagation medium was limited to water. However, the simulations could, in principle, be performed in other media, such as blood or biological tissues, for which the frequency-dependent acoustic attenuation is much higher.^34^ This will result in further attenuation of especially high-frequency components of ultrasound upon propagation to the focus and, thus, to a reduced focal gain. Unfortunately, the axisymmetric model cannot be used for these more relevant materials due to limitations in the supported attenuation models.^15^ Simulations in blood and other media could be performed with full 3D simulations, but these are computationally expensive; performing parameter sweeps, such as those presented in Fig. 8, would, therefore, take orders of magnitude longer. Nevertheless, performing a single full 3D simulation (medium properties, $c_{0}=1590\;\;m/s,\rho_{0}=1049$ kg/m^3^, $\alpha_{0}=0.0546\;\;\text{dB}\;\;{\text{cm}}^{-1}\;\;{\text{MHz}}^{-b}$, *b* = 1.58, and B/A=6.1;^21,35^ data not shown) confirms that peak negative pressures that are sufficient to induce free-field cavitation in the blood ($P_{-}$ threshold = –26.9 MPa^27^) can be obtained with a LGFU transducer that has a diameter of 3.5 mm, a radius of curvature of 2 mm, and a maximum pressure at the transducer surface of 9 MPa. Thus, our model predicts that therapeutic effects can be achieved, even in the blood, using transducer geometries suitable for interventional instruments.

## VIII. CONCLUSION

Through a series of simulations and experiments, we aimed to answer whether miniaturized LGFU transducers could achieve sufficient pressure at the focus to induce intrinsic cavitation. We developed the first extensive numerical model to predict the capabilities of LGFU transducers. The framework is based on the *k*-Wave toolbox, which allows for simulations in both lossless and absorbing media as well as linear and nonlinear propagation.^36^ To significantly increase simulation speeds, an axisymmetric coordinate system was implemented, which reduces the total number of the grid size from 3D to 2D.^15^

As correctly defining acoustic source terms is a crucial part of numerical simulation in ultrasound, particularly in therapeutic ultrasound, to make accurate predictions, several measures were taken. First, staircasing errors were prevented by representing the transducers by an off-grid acoustic source distribution.^16^ Second, the model allowed for arbitrary radially symmetric illumination patterns to accommodate real-life light delivery scenarios to avoid overestimation of the achievable pressures. An unrealistic case of uniform light delivery was shown to overestimate the pressure up to 1.8 times higher compared to a realistic counterpart of diverging light delivery. The flexibility of the model in the source distribution was applied to a novel scenario that achieved significantly higher pressures (35% increase was observed in the experiments) than conventional collimated illumination.

The model was validated against experimental data for a range of geometries, illumination scenarios, and linear and nonlinear regimes. Therefore, the model can serve as a tool to determine the transducers' performance without elaborate experiments and be guiding for inaccessible media such as blood and soft tissue.

For the more attainable case of diverging light delivery, the model was used to confirm that free-field cavitation can be achieved using LGFU transducers with a diameter as small as 3.5 mm. Transducers with these small lateral diameters can be integrated into interventional instruments such as endoscopes and steerable catheters. Interventional LGFU, thus, shows great promise for future therapeutic applications in interventional surgery.
